# Social Capital and Social Networks of Hidden Drug Abuse in Hong Kong

**DOI:** 10.3390/ijerph17176231

**Published:** 2020-08-27

**Authors:** Gloria Hongyee Chan, T. Wing Lo, Gabriel Kwun-Wa Lee, Cherry Hau-Lin Tam

**Affiliations:** Department of Social and Behavioural Sciences, City University of Hong Kong, Hong Kong, China; glo.journal@gmail.com (G.H.C.); gabriellkw@gmail.com (G.K.-W.L.); ss.hltam@cityu.edu.hk (C.H.-L.T.)

**Keywords:** hidden drug abuse, drug trends, social capital, social networks, Hong Kong

## Abstract

Owing to the increasing prevalence of hidden drug abuse in Hong Kong, yet scarce relevant current local research, this study seeks to carry out an in-depth investigation into the experience of hidden drug abusers, paying particular attention to their relevance to social capital and social networks. Seventy-three abusers attending drug treatment programs were interviewed, and a thematic analysis was performed. The results indicate hidden drug abuse is popular in Hong Kong. Apart from the decline of public, large-scale discos and the change of the types of drugs abused to date, one important contributing factor is the drug supply and transaction networks, which are extensive and multilocused, but secretive, with high closure levels. This kind of network is supported by bonding, bridging, and linking social capital as well as by providing convenient supply modes and offering drug abusers psychological comfort and safety. These factors encourage the hidden drug abuse to prevail and allow drug abusers to remain unidentifiable.

## 1. Background

### 1.1. Drug Abuse in Hong Kong

The number of first reported drug abusers in Hong Kong has consistently decreased from 2029 in 2016 to 1544 in 2019 [1]. This decrease does not, however, indicate that the drug abuse phenomenon is less severe, it rather reflects the increasing difficulty in identifying drug abusers due to the increasing prevalence of hidden drug use [1,2]. As early as 2008, according to the government Task Force on Youth Drug Abuse, users’ homes were the most common venues for drug abuse among youth; furthermore, traditional drugs (e.g., heroin) were replaced by psychotropic drugs, whose withdrawal symptoms were slowly emerging, and little equipment was required for taking such drugs [3]. This constituted the “hidden nature” of drug abuse [3] (p. 18), making immediate detection of drug abuse cases challenging.

Hidden drug abuse in Hong Kong is not a long-standing phenomenon; rather, it is a result of the change in drug-taking patterns. Because of police crackdowns on drug activities at discos, large-scale rave parties started to vanish and were replaced by the underground rave culture—small-scale, less structured underground activities, scattered into various places, such as privately-operated parties, resort houses, units in industrial buildings, karaoke bars, and even shopping malls during non-business hours [4]. Drug-taking activities took place in three kinds of situations: (1) in “organized, structural, and commercialized settings, such as small-scale discos/dance clubs”; (2) in “spontaneous and self-initiated ways”, which were often in-group or peer-related, in various places such as the youth’s own homes or their friends’ homes, karaoke bars, video game centers, or parks; and (3) as a form of entertainment in addition to other activities [4]. Hence, drug-taking behavior occurred everywhere, including at beaches, at podiums of public housing estates, and at cinema houses/movie theatres. According to the government Narcotics Division [1], of the various drug-taking activities ranging from private (e.g., homes, resort houses, and hotels) to public (e.g., discos/karaoke lounges, cyber cafes, and video game centers), youth’s own homes and their friends’ homes have been the most popular (52.9% in 2016 and 54.4% in 2019).

Since 2007, psychotropic drugs such as methamphetamine and cocaine have become more popular than traditional drugs or narcotic analgesics (e.g., heroin) [1,5]. Among all drug abusers who are first reported, nearly 90% of them take psychotropic drugs (88.8% in 2016; 90.3% in 2019). The change to psychotropic drugs leads to a more complex and dynamic drug market due to the unfit epidemiological indicators for monitoring the new substances [6]. Furthermore, since psychotropic drugs are less likely to cause withdrawal symptoms, abusers are difficult to be identified, even by the closest family members, until the abusers suffer from severe side effects [7], such as deteriorated judgment and memory, heart disease, and kidney failure [8]. Psychotropic drug use causes hallucinations, which trigger drug abusers to develop self-defense mechanisms and become socially isolated [9]. This suggests that the unique features of psychotropic drugs abused to date also contribute to hidden drug abuse.

Despite the prevalence of the problem, the hidden nature of drug abuse has been under-researched in this region. The present study seeks to uncover the operation networks of hidden drug abuse in Hong Kong. In particular, it investigates how the hidden drug abuse networks are sustained. Since networks involve many people, the concept of social capital is used as the framework of analysis.

### 1.2. Social Capital and Drug Abuse

Social capital is commonly referred to as resources that are embedded in social networks that people access in order to meet different needs. Social capital can have particular advantages and disadvantages [10,11,12,13]. Social capital encompasses (1) the nature of networks (e.g., norms, obligations, rules, reciprocity, and trust) that help individuals to attain their goals [14,15] and (2) the structure of networks, such as bonding, bridging, and linking social capital. As one type of social capital, bonding social capital is the development of networks that are formed by groups with a high degree of homogeneity, such as family members and close friends who have a similar socio-financial position, demographic characteristics, and shared identities. For another type, bridging social capital involves the formation of networks by people who do not share the same demographic background, but who have similar financial and power statuses. The final type, linking social capital, involves networks formed by people across institutions [15,16,17], whose formations facilitate the acquisition of resources. Through personal investment in establishing and maintaining social networks, individuals are able to obtain social capital directly (i.e., from socializing with people) and indirectly (i.e., acquiring the extended resources brought about by the social capital obtained) [18,19].

Meanwhile, social capital cannot be understood independently from the broader institutional environment, so the power difference existing in the social networks should not be neglected when understanding social capital [15], which prefers a dynamic, rather than a static, perspective. Combining the three types of social capital together—bonding, bridging, and linking—allows for an optimally constructed social network that varies according to the needs and priorities of the community and the macro-environment [16]. This optimally constructed social network contains horizontal relationships (e.g., bonding and bridging social capital) and vertical components (linking social capital) [12,16]. Therefore, taking into consideration the three types of social capital offers a comprehensive understanding of a particular social network.

Furthermore, social capital has been regarded as an important factor in health [20]. Some scholars have said that having high levels of social capital helps people have less unhealthy behavior, such as addictive behavior, because it helps them obtain more social support at times of stress [21,22], which helps them not to resort to substance abuse as a way of coping [20]. In addition, social capital helps facilitate the exchange of health-related information, the indoctrination of prosocial norms, and the provision of resources that encourage prosocial behavior [20,23].

However, social capital does not always guarantee reduced substance abuse; rather, it can increase people’s risk of engaging in substance abuse [24]. It is mentioned that having drug abusers in one’s social networks can lead to increased access to social capital related to deviant and even criminal acts (e.g., illegal drug transactions), and this can in turn affect the individual’s acquisition of conventional social capital [25,26]. It is further illustrated that sociometric networks can be related to drug abuse [25]. Sociometric networks are referred to as the entire set of personal relations, encompassing direct (i.e., people with whom one has direct interactions) and indirect (e.g., friends of friends) ties [27]. As long as individuals are connected to a network that includes drug-taking members, they become part of the sociometric network of drug abuse. Making direct contacts with drug abusers increases one’s opportunity of making contacts with other drug abusers through these contacts [25]. Through chains of direct and indirect networks, the transmission of drug-related knowledge is facilitated [25]. Thus, it is important to take note of the nature and structure of social networks, as they can exert different effects on the likelihood of drug transactions.

To look at how social networks influence drug abuse, one can make analyses based on the structure and functions of the networks [28]. While the networks’ structure encompasses the nature of the members, the networks’ size, people’s position within them, and the networks’ density or cohesiveness [29], the networks’ function is referred to as “the roles that network members play” [30] (p. 2), in which norms operate as important tools for social regulation and influence [31].

Regarding the structure of networks, to begin with, the size of an individual’s networks (i.e., the number of direct ties that the person possesses and maintains) influences that person’s tendency to engage in drug abuse [29,30]. Since members within a network likely influence one another through socialization [32,33], having a larger proportion of drug abusers in a network likely encourages the others’ drug abuse [34]. Next, an individual’s position in the networks is also an influential factor of drug abuse [29,35]. Members who are in the central position of the networks, regardless of direct networks or sociometric networks [29], have more “linkages within that network and consequently are more active, in comparison to peripheral actors” [30] (p. 3). In this sense, those who are in the core position, having a higher status in the drug networks, are likely to be more agreeable to the drug-related norms and thus more engaging in drug abuse [30], and they are more influential to other members [36]. On the opposite end, peripheral members in the networks are likely to have more access to other sources of information and resources, and are thus less subject to the influence of drug-taking members in the networks [37].

Moreover, a network’s density or cohesiveness plays an important part in influencing its members’ drug abuse [29]. A person who is more connected to a group is likely to experience more sanctions from the group if that person deviates from the norm [30]. In this sense, if a person is embedded in a dense, closed nature of drug networks, he or she is more likely to internalize drug-related norms. Such culture thus becomes self-reinforcing and less accepting of new, alternative behavioral patterns [30,38,39]. To summarize, networks containing more drug-taking members, especially those with high levels of cohesiveness, are more likely to encourage drug abuse through the accumulation and transmission of drug-related social capital. The nearer people are to the center of drug abuser networks, the higher their level of susceptibility to the influence of their drug-taking peers’ drug-related norms.

Moreover, it has been contended that criminal networks usually have fluid forms, are less hierarchical, and have looser connections between members. This enables the networks’ capacity to adapt to environmental changes flexibly and to become more resilient [40,41]. Additionally, such networks’ relative “lack of physical infrastructure” means that they “can relocate operations from one geographic area to another” and thereby reduce the risk of being detected by legal institutions [40] (p. 239).

**Figure 1:**
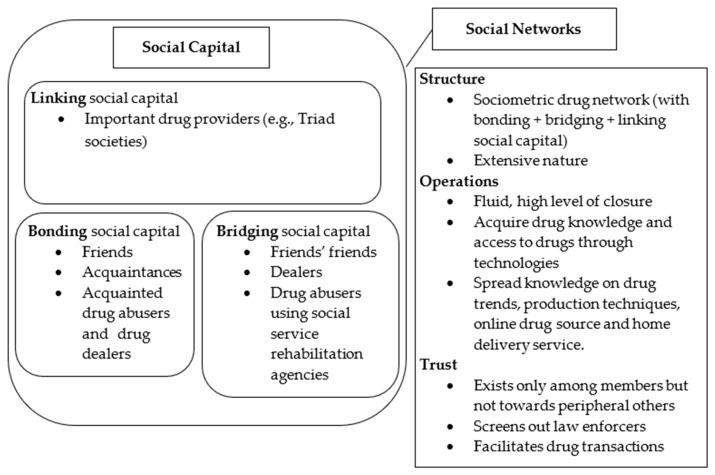
Social capital and social networks of drug abuse.

Guided by the literature, the present study adopted a social capital framework (see Figure 1) to investigate (1) the changing trends of drug abuse in Hong Kong, especially its hidden nature; (2) the social capital embedded in abusers’ social networks; and (3) the operating mechanisms of the drug abusers’ networks. According to the Task Force on Youth Drug Abuse [3], drug abusers’ homes, friends’ homes, and other private venues with access control are considered concealed and less organized venues for hidden drug abuse. In the present study, hidden drug abuse is defined as drug-taking behaviors that are performed in private and access-restricted venues.

## 2. Materials and Methods

### 2.1. Recruitment and Samples

Participants in this study were former drug addicts detained in four correctional institutions for drug abusers. They attended addiction treatment programs under the Drug Addiction Treatment Centres Ordinance (Cap, 244). There are only four statutory correctional institutions in Hong Kong that run drug addiction treatment programs, and all of them were included in the present study. The purposive sampling method was adopted. A total of 73 inmates participated in this study (see Table 1). Half of them were men or young men (50.7%), while the other half were women or young women (49.3%). Their ages ranged from 11 to 70 years, of which 58.4% were aged below 30 years. In addition, more than half of them (64.8%) used to take drugs more than six times per week. Crystal methamphetamine (also known as ice or crystal meth) was the most popular type of drug taken (67.1%). Regarding the participants’ experience with drug abuse, most of them started this habit when they were between 11 and 20 years old (78.3%), and half of them took drugs for more than 10 years (50%). Regarding the venues for drug abuse (see Table 2), the most significant place was their homes, either their own (56.2%) or their friends’ homes (20.5%). This factor reflects the conclusion that hidden drug abuse among them was prevalent.

### 2.2. Data Collection and Analyses

This study adopted a qualitative research methodology. After the research was approved by the institutional review board of City University of Hong Kong, the researchers invited all the inmates who had been admitted to the four correctional institutions aforementioned between February and April 2017 to participate in the interview. Written informed consent was obtained from all willing participants before the interview. Each of the participants took part in a 30-min to one-hour semi-structured interview, in which their experience and views of drug abuse were obtained. The four authors themselves, not research assistants, conducted face-to-face interviews with the drug abusers. All the interview data were transcribed into written Chinese. Thematic analysis was adopted to capture the experience regarding hidden drug abuse, in the light of social capital and social networks, based on the insights from the literature.

Following the six coding steps of Braun and Clarke [42] (pp. 87–93), the authors adopted line-by-line coding to familiarize themselves with the data. Once the initial codes and primary themes were generated, the authors consistently reflected on and reviewed the original data and generated codes to ensure consistent reflexivity. Finally, the codes were derived from the data and linked to form meaningful themes and sub-themes for analysis (see Table 3). A second coder was invited to code 20% of the participants (20.5%, *N* = 15) independently as a reliability check. The inter-rater reliability was computed by ReCal2 [43], and Cohen’s kappa ranges from 0.804 to 1 were obtained. Cohen’s kappa of 0.80 or greater was recommended to be accepted in all situations [44] (p. 168). Consensus agreement for the differences in the codes was achieved after the discussion between the two coders. All quoted data were translated into English by one of the authors and checked by another author to maintain accuracy.

## 3. Results

### 3.1. The Current Trend of Drug Abuse

#### 3.1.1. Switch from Large Discos to Private Parties

Echoing the findings of previous research [9], participants (*N* = 7) perceived the decline of discos as contributing to the aggravation of hidden drug abuse (e.g., HS012). HS006 said, “In the past, I went out once or twice a week for taking drugs. Now the discos no longer exist, so I would take drugs at home with friends every day.” Another participant, HR001, revealed that the active police raids drove him to take drugs at home: “These days, the police have been proactive, and they patrol everywhere. It made me stay at home to take drugs more often.”

The drug networks are of an elusive nature, with widespread hidden locations. Private parties became the most popular venue after the closure of discos (*N* = 9). HR004 disclosed that “the private parties are everywhere, having existed for at least eight to ten years.” LSF001 added that “there are at least 20–30 private parties. They are situated anywhere you can ever imagine, like industrial buildings and ordinary buildings, in Tsim Sha Tsui and Mong Kok. You wouldn’t even imagine that you could get ice (crystal meth) from a game machine; it is in an apartment in Mong Kok.” This shows that hidden drug abuse is pervasive, extensively developing in multiple locations.

Despite private parties’ long period of existence, it appears that most average people are rarely aware of them. HI009 echoed other respondents, saying that there were many private parties for taking drugs, “however, the police cannot find them. Few cases have been reported.” LSS003 added that private parties were difficult to discover: “These places are not doing business openly; they are behind closed doors. Before you can enter these places, you must be initially identified via a closed-circuit television system.” This statement indicates that private parties have a much higher level of privacy than large discos. Thus, sweeping out the discos did not effectively eliminate drug abuse; rather, it drove abusers to resort to private venues for taking drugs.

#### 3.1.2. Availability of and Accessibility to Drugs

According to some participants (e.g., HF002, HS011, and NR002), abusers could easily find drugs across the 18 districts of Hong Kong. Acquainted abusers definitely know the drug transaction locations (HR007). They can easily locate, within a single district, twenty to thirty drug selling points (LSF001), which are unrecognizable by non-abusers because they appear normal and inconspicuous, such as small apartments in public housing estates and industrial buildings. Hence, ordinary people may not be able to realize that there are nearby drug selling points, even if there is one in a familiar building (HF001). The locations have high levels of secrecy (*N* = 12). However, HR007 mentioned that “drug abusers must know these places”, which suggests that although the locations are unknown to the public, drug abusers who are within the circle come to know them through their own drug networks. Moreover, while the drug supply networks exist in different localities, the transactions can be cross-district. For HR007, a user-dealer, his drug-taking clients came from everywhere: “At first, it was my friends who introduced clients to me. Later, it was my clients who introduced me to other clients who came from other districts. So, I had cross-district transactions, too.”

Several participants mentioned the extensiveness of drug supply networks (*N* = 24). Drug supply networks extended to every possible location (*N* = 13), such as housing estates (e.g., NS012, LKR001), schools (HI010), and gaming centers (e.g., HI009). One participant (NI009) mentioned the “drug canteen”: “Actually buying and getting drugs is convenient. Some people love to take drugs right after getting them there. That place is what we call the ‘nest.’” NI002 further illustrated the wide availability of drugs, saying: “You simply can’t miss the drug supply networks because they’re everywhere. Maybe there are already some of these people living in your building. Even if the police arrest one of them, there’ll be another one.” These accounts reflect that the drug supply networks are boundaryless, and drugs are easily accessible.

Regarding locations, the participants often mentioned public housing estates (e.g., HR004, HI009, HS005) and squatter areas (e.g., HS011). For example, HI008 mentioned his connections in a public housing estate in Tuen Mun: “I met dealers in the streets when I was young, and they became my friends. It can be said that they’ve taught me things related to drugs.” NI003 mentioned that he lived in a housing estate in the Northern District, a relatively underprivileged neighborhood, so it was easy for him to take drugs from friends. This suggests that the drug supply networks are highly proximal and easily accessible. Once a person gets in touch with drug-taking peers within the same neighborhoods, the networks then expand based on similar interests.

Intriguingly, since rehabilitation and social service agencies assemble abusers, dealers take such opportunities to sell drugs outside the service centers, thus making these service places focal points for drug transactions. For example, HS002 said, “Outside the Methadone Centers, one can find a drug supply. If you want to buy drugs, you are able to get them there.” HS010 added, “I heard the social service agency would invite ex-drug addicts to live in the hostels for rehabilitatees. However, drug transactions are taking place downstairs on the street corners.” This indicates that the drug supply networks extend and permeate into broader levels of community social systems.

#### 3.1.3. Psychological Safety When Taking Drugs Privately

Several participants expressed the changes in the types of drugs taken today, which may contribute to hidden drug abuse. For instance, NI010 indicated that the decline of discos resulted in his change in the choice of drugs: “When I was in discos, I took ecstasy and ketamine. Now, when I started to take ice, I took it at home.” Likewise, as explained by HF002, “In the past, ketamine and ecstasy were popular, now people have turned to cocaine and ice, which they take at home. The drug market has changed. It’s the type of drugs that matters. In the past, taking ecstasy was not obvious. How would other people know about it? But now, people take ice and they need tools to take it, so they need to hide.” LSR001 echoed this belief, saying that some drugs were easier to take (e.g., ketamine), while some were more difficult: “You need tools to take ice, but you can’t always have them with you; they are at home.” This shows that the types of drugs taken today contribute somewhat to the choice of venue for drug abuse. Moreover, despite the obvious withdrawal symptoms found in heroin-users, some participants mentioned that there were less obvious signs of psychotropic drug taking. HS008 explained, “You can’t tell if somebody takes drugs or not, when they dress so decently.” All of these accounts show that the nature of drugs taken may contribute to drug abuse not being discovered.

For some participants, taking drugs alone in private was more comfortable: “If I could stay at home to take drugs comfortably, of course, I would do so. I felt uncomfortable when taking drugs, which might be contaminated, and taking drugs with many strangers outside” (NI009). Some participants felt psychologically safer because some drugs create hallucinations (e.g., LKS001), which led them to avoid others. NI009, for example, preferred taking drugs alone: “I have experienced hallucinations. At that time, I took drugs with my friends, you would feel that they were saying something bad about you, but in fact, they said nothing. When I was living with my boyfriend, as he took drugs, too, we both had hallucinations, which caused us to have serious conflicts.”

In addition, some participants mentioned that they took drugs in private because they did not want their drug-taking habits to be known to others. For example, HI008 mentioned that taking ice freed him from meeting other people: “I feared that other people would know my past, so I wouldn’t meet new friends. Taking ice, I didn’t need to face others and could stay at home hidden.” NI010 also mentioned that he did not want other people to know about his drug-taking habit, because “this is not a good thing”, implying that he was worried about being stigmatized and discriminated against due to his identity as a drug abuser [45].

For some participants, taking drugs at home was psychologically safer due to no more fear of being arrested (e.g., HI002 and HF001). LSS003 explained, “If you keep on taking drugs publicly, you’ll be afraid of the police.” HS013 added, “How can the police arrest the drug abusers if they’re not on the streets but taking drugs at home, unless they’re discovered by their families and reported to the police.” HI004 expressed her belief that the more active government actions to wipe out discos would induce hidden drug abuse: “Originally, there were places for taking drugs together, now the discos are gone, so we were like hidden youth, taking drugs at home. It’ll definitely cause a change. If the government wishes to crack down on drug abuse, they need to target hidden youth.” This indicates that taking drugs hiddenly is not only more comfortable for the abusers, but may also be a strategy for them to protect themselves from getting arrested. The consequence of hidden drug abuse was that the identification, detection, and combat of drug abuse would become increasingly more difficult (*N* = 8).

### 3.2. The Structure of Social Networks and Social Capital

#### 3.2.1. Drug Abusers’ Social Networks via Bonding and Bridging Social Capital

Social capital is embedded in social networks. A social network for drug abuse that is constructed by different forms of social capital leads to a convenient way to obtain drugs. Social capital facilitated drug abusers to transmit and exchange information in their social networks, which had a sociometric and extensive structure, as well as fluid and closure operations. One source of getting drugs was through personal connections—obtaining drugs from friends directly or indirectly (*N* = 13) (e.g., HS006, HI002, HI003, NI009). For example, HS011 contended that “friendship is the only way to establish the connections and network”. HR003 obtained drugs from drug-taking friends he knew when he was a teenager. Getting drugs through friends can be regarded as part of bonding social capital [46]. Some participants (HS010, HS011, and HS003) explained that they obtained drugs through personal networks, such as through friends and acquaintances who were introduced by friends and acquaintances, which is bridging social capital.

Some drug abusers were also drug user-dealers (*N* = 23) who provided drugs for themselves and for their friends. Being drug user-dealers was profitable (e.g., HI003, LSF001) because it helped them cover the cost for drugs (e.g., HS011, HF001) and sustained their drug-taking habit. HR004 said, “The money was enough to help save the cost for buying drugs. The most important thing is not to suffer loss.” User-dealers explained that such a way of making money was normal because they understood the rule of supply and demand. This belief is reflected in what HS009 said: “Yes, there are demands so there are supplies. If no people take drugs, why would people buy drugs?... Even if you don’t sell drugs to your friends, they will simply turn to another dealer for drugs… You actually can’t stop the drug transactions.”

A few participants reported that they made drugs for themselves and for their friends (e.g., LSF001). HF002 said, “Ice is cheap because everyone can make it themselves now! Just surf the Internet and read the teaching materials.” NR005 mentioned that her boyfriend made drugs himself and gave her drugs every day for consumption. NI010 also made drugs for his own use: “I’ve found a better method for taking ice. There are many things you can just simply do by yourself. Home-made stuff is better, of course. So, I began to stop taking drugs in public, but [instead took them] in my own place… alone.” Self-containment reduced the need of taking drugs with other people or of going to parties to get drugs. Such convenience further fosters the hidden nature of drug abuse.

#### 3.2.2. Drug Supply Network via Linking Social Capital

Triad society plays a dominating role in the Hong Kong drug market [47]. More than ten participants (e.g., HR004, HS008, HS011, and HS013) identified that they had connections with Triad societies, which provided advantages for them in drug transactions. For instance, HR004 took drugs after becoming acquainted with Triad members who brought him to discos and provided him with free drugs. LSF001 mentioned that he obtained drugs from a Triad leader whom he followed: “The Triad leader supplied me drugs, then I sold them to other people and made a profit from it. It’s a reciprocal relationship, in which both of us got what we wanted.” The connection with Triad members was believed to be a factor for the abusers’ first-time drug use due to their Triad lifestyle, such as seeking thrills and adventure (e.g., HI009 and HR004). HR004 added that taking drugs with Triad members was a means of avoiding being alienated in Triad societies.

In addition, Triad members also helped transmit the knowledge, methods, and techniques of drug-taking (e.g., HS008 and HS011). Therefore, the participants’ associations with Triad members helped expand their connections with user-dealers so that they could exchange drug information, guarantee a regular drug supply, and obtain cheaper drugs easily (HS003, HS005, LSS002, and LSF001). For instance, HS005 relied only on the supply from a Triad society to support his persistent drug abuse, so he did not need to seek drugs from other sources. The drug supply from Triad societies was even sufficient for the participant to sell to other people to cover the cost of drug-taking (LSF001), thus sustaining his addiction. Moreover, some Triad members are also drug abusers themselves (LSF001), hence there is both supply and demand of drugs in the Triad underworld. Therefore, associating with Triad societies sometimes became an advantage. However, as disclosed by one participant (HS003), because linking up with Triad societies was a means of obtaining a regular supply of drugs, there was no need to engage in other Triad criminal activities.

#### 3.2.3. The Mechanisms of Drug Transactions

The drug supply network operates across different districts in Hong Kong mainly by home delivery services and by hand-to-hand transactions. With the aforementioned availability of drugs in various districts, geographic boundaries are no longer a hurdle to the drug business (HS002). HR005 and HR007 pointed out that even if they could not obtain drugs in their own communities, they could easily obtain drugs cross-districtwise.

Some participants mentioned that they ordered drugs through home delivery services (*N* = 7) (e.g., HI010, NS007). Such delivery has been fostered by technological advancements. The platforms for communication included mobile phones (*N* = 7) (e.g., HI004; HF001) and the Internet (*N* = 1). For example, HS010 mentioned that drugs could be obtained by simply calling someone, while NI009 said, “I’ll use WhatsApp. Now it’s technologically advanced. WhatsApp is convenient to use.”

Some participants mentioned buying drugs outside social service and rehabilitation centers (HF001, HS002, HS007, HS011), whereby they also shared information and ways to access the supply networks in other districts. Exchanging telephone numbers among the abusers was the most typical way to maintain connections (HF002). Some participants mentioned that they obtained drugs from friends, meeting them in person at certain places and getting the drugs in a hand-to-hand manner (*N* = 3). As explained by HS011, this practice helps share the liability: “If doing the transaction hand to hand, we’re both guilty if arrested. But if the dealer puts the drugs into the planter and then I go to collect them, the police can arrest only me.” The above accounts indicate that drug dealers and abusers in the networks maintain connections with the aid of telecommunications, and the transactions of drugs involve a fluid supply structure.

#### 3.2.4. Trust as the Key to Enter the Networks

Social capital values the information exchange, cooperation, and even trust associated with social networks. Due to the secret nature of a drug abuser’s network, trust is essential for a stranger to access the network. In the present study, although the trust identified was low-level trust (discussed below) as distinguished from the high-level trust commonly practiced in organized crime [48], it is still the key to enter the network of drug abusers.

Some participants only transacted drugs with acquainted abusers and dealers (e.g., HS013, HF002), where trust was key for the transactions (LTCS007). The punishment for drug trafficking is severe, so dealers used all means to reduce the risk (HR004), where trust is essential to assess the identity of customers and to avoid undercover police (HF002, HS011, LSF001). Drug transactions with acquainted people enhanced security and managed risk because the places for transactions were only known to the acquainted members but not to peripheral others (HR006).

Since trust is so important, some participants approached the drug suppliers with the referrals and introductions from friends before they were eligible to obtain drugs from the suppliers (HS007, HS009, HS010, HF001, HR004). Participants also explained that a discounted price would be offered by suppliers to the acquainted user-dealers (HF001, HI006). Once drug transactions were established between the known persons (HF002), the user-dealers could sustain their business networks (HF001, HR002).

Moreover, drug dealing through acquaintances brings a stable source of customers. According to HR007, “When abusers get used to obtaining drugs from you, they will only buy drugs from you rather than through other dealers.” Similarly, HS005 and HS006 both relied on acquainted persons to obtain drugs without ever trying other means. Based on the element of trust, NI009 relied only on the established network of customers to operate his drug business, which provided sufficient income to him.

Similarly, referrals and introductions from acquainted abusers are necessary for strangers to get access to private drug parties. Coherently, NI010 stated that there was access control in private parties in which security guards allowed the entry of acquainted and trusted persons at night time. LSF001 and HS008 mentioned that strangers could never get into these private parties even if they rang the doorbell. LSF001 illustrated the operation of the private parties, saying: “There are Closed-Circuit Television systems installed there, and people are monitoring. Only people with close relationships can get in. Even if a stranger knew this place and pressed the doorbell, they wouldn’t open the door for you.” This implies that only allowing close, trusted people to get into the hidden spaces helps ensure safety and avoids any risks of being discovered by the police [24].

While trust is difficult to establish without referrals and introductions, connections develop immediately once rapport has been established. LSS002 noted, “Once you’ve entered this particular social circle, then some people may get to know you. They’ll tell you they have drugs. Then when you are in need, you can just call them for drugs. You’ll just keep on knowing people in the parties, so you’ve many means for getting drugs.” Within this closed but fluid network, information can be transmitted quickly. Other abusers and dealers can easily be recognized. NI009 disclosed that “in this circle, you’ll get to know more and more of these people quickly. This means we’re all the same—drug abusers.” Similar situations occurred for drug transactions between abusers hanging around outside social service and rehabilitation centers because the status of the customers as drug abusers could be easily verified.

## 4. Discussion

As mentioned, social capital refers to the resources embedded in networks, in which networks of a particular nature and structure can more likely be mobilized for meeting individual needs [11,14,17,46]. Social capital is important for abusers to obtain drugs in horizontal (e.g., friends and friends’ friends) and vertical (e.g., Triad society) manners. Drug abusers turn not only to friends (bonding social capital), but also to dealers and even friends’ friends (bridging social capital) to obtain relevant information and to gain access to drugs [7,49]. Hence, drug abusers live in a chain of sociometric networks containing both direct ties and indirect ties with other drug abusers and dealers [25,27].

Although the participants revealed few clues about their positions and statuses in the networks, it is known that getting to know Triad societies (linking social capital) guarantees the supply of drugs. The existence of a power difference between Triad societies and user-dealers constructs a linking social capital in the drug supply network, even if the user-dealers are not fully embedded in Triad activities. Drug user-dealers can rely on the supply of drugs from Triad societies to make a profit to sustain their drug habit. This is an example of the type of acquiring economic capital through social capital that Bourdieu [50] explained as the interchangeability of the four forms of capital, including economic capital, social capital, cultural capital, and symbolic capital. Furthermore, it appears that regardless of the level of cohesiveness of the drug abusers’ networks, solely being embedded in the sociometric networks can sustain drug abuse as the source of drugs and the exchange of drug-related information can be secured. This is consistent with previous research, which stated that linking social capital can become nepotistic or a mechanism for insider-trading without proper control and accountability [12]. Combining the three types of social capital with a dynamic social network of hidden drug abuse can be identified.

Drug abuse and drug dealing have their own networking patterns, which help sustain such activities [41]. Unlike highly organized networks of drug trafficking [51], in the context of the former drug abusers in the present study, the drug supply networks appeared to be fluid, loosely connected, non-hierarchical, and without physical infrastructure, as other scholars have mentioned [40,41]. Moreover, drug abuser networks allow the entry of members through the snowball effect (e.g., friends introducing their friends and acquaintances) [40]. The networks of the drug abusers are far-reaching, not only developed based on certain geographical communities, but also disseminated based on common interests in drug transactions, which serves as a functional community that helps delimit the restrictions of space [52]. Despite the snowball effect means of expanding the networks, the opportunity of being infiltrated by the police is minimized because the networks are formed in disperse locations yet in extreme secrecy and close membership. This reflects the exclusive nature of social capital. Social capital is not always inclusive, as Coleman [11] explained it—a public good in which the resources in the networks can be accessed by every member and can benefit all. It is, to the contrary, exclusive. The transmission and exchange of social capital fosters “mutual acquaintance and recognition” among members in the same group only [10] (p. 119), and members delineate themselves from other groups and classes [50]. With their highly secretive nature, such networks remain invisible to the public eye, thus increasing the difficulty of crackdowns on drug abuse.

In addition, it is noted that the drug supply networks have been facilitated by the Internet. The source for obtaining drugs and drug-related information is convenient [7,49]. Alongside technological advancement, the Internet has become a source of information about the drug trade [53,54] and a platform for reaching hidden abusers [55]. Some websites provide information about the procedures for manufacturing drugs, suggesting that drug abusers can become drug manufacturers or distributors as well [56]. The self-manufacture of drugs further contributes to the difficulty of regulating and monitoring the drug markets because the substance and epidemiological indicators are unknown to the authorities [6]. Serious harm or even fatal poisonings can probably occur while using self-manufactured drugs [6]. Moreover, the Internet serves as a marketplace that allows online shopping for drugs [45,57,58]. As such, the Internet has become a significant space for facilitating drug dealers’ business [55,58,59]. The Internet also facilitates buyers’ purchasing drugs online, as sources even deliver them through home delivery services, further fostering hidden drug abuse.

The present study identified trust as a determining factor for attaining access into drug abuser social networks. As Yeşilyurt [41] put it, to protect members in the networks from potential harm, “trusting the right person is the key” (p. 287). Trust has also been found to be a key in organized drug trafficking networks [51] and the familial illegal trading networks [48]. In the present study, trust, albeit at low levels, functions to allow an abuser to enter into networks and to acquire social capital. However, the networks are fluid, and the ties and trust relationships are not as rigorously controlled and monitored as they are in traditional organized crime [47]. Trust is established through referrals and introductions from the friends, clients of drug rehabilitation centers, and Triad members. This helps to screen out law enforcers and to distinguish peripheral others from abusers during drug transactions [24]. Trust, hence, is an integral part of the network.

## 5. Conclusions

The results uncover the experience of drug abusers in Hong Kong. While the participants acknowledged that the decline of public, large-scale discos, the result of proactive police intervention, was as an important push factor driving people to take drugs privately, there are also pull factors attracting people to take drugs hiddenly. Apart from the perceived feelings of security and the changing drug trends that require different drug-taking methods, the nature of drug abusers’ networks is also a noteworthy factor that contributes to the sustainment of hidden drug abuse. This finding supports existing literature that indicates that social networks are a “key to understanding” drug abuse [30] (p. 2). The concepts of social capital and the features of networks affecting the engagement in drug abuse as reviewed in the literature can be applied to understand the phenomenon of hidden drug abuse in Hong Kong.

The results support the claim that social networks that contain drug-taking members will encourage and reinforce drug abuse [34] (see Figure 1). Although social capital exists in social networks, social bonds do not guarantee information flow that would help prevent drug abuse [20,60]. Rather, they can also encourage the ongoing occurrence of drug abuse through offering ways to obtain drugs and exchange drug-related information in the underworld [60,61]. Furthermore, the Internet serves as a bridging social capital that links drug buyers and dealers who are not necessarily friends or acquaintances. The Triad societies also serve as linking social capital that constantly supplies drugs. They foster the circulation and exchange of drug knowledge and information, thus further sustaining drug transactions and abuse. Hidden drug abuse appears to remain a prevalent issue in Hong Kong. A probable result of prolonged hidden drug abuse is the severe and irreversible harm to users’ physical health by the time they seek help or are reported. Policy makers should consider this issue proactively and find ways to enhance the early identification of hidden drug abusers and provide the appropriate treatment accordingly.

## Figures and Tables

**Table 1:** Participants’ demographic and drug use data.

Variable	No. of Participants (*N*)	%
Gender (*N* = 73)		
Men	37	50.7
Women	36	49.3
Age (*N* = 72)		
11–20	13	18.1
21–30	29	40.3
31–40	17	23.6
41–50	9	12.5
51 or above	4	5.6
Frequency of taking drugs (*N* = 71)		
Less than once per month	1	1.4
Once per month	1	1.4
2 or 3 times per month	6	8.5
1 or 2 times per week	10	14.1
3–6 times per week	7	9.9
More than 6 times per week	46	64.8
Types of drugs taken (could choose more than one option)		
Crystal methamphetamine (Ice)	49	67.1
Cocaine	26	35.6
Ketamine	24	32.9
Heroin	16	21.9
Nimetazepam	10	13.7
Cannabis	8	11.0
Ecstasy	7	9.6
Others (triazolam, methaqualone, cough medicine)	8	11.0
Age at first drug taking (*N* = 69)		
11–20 years old	54	78.3
21–30 years old	10	14.5
31–50 years old	5	7.2
Duration of taking drugs (*N* = 72)		
Fewer than 3 years	14	19.4
3–5 years	10	13.9
Between 5 and 10 years	12	16.7
Between 10 and 20 years	23	31.9
More than 20 years	13	18.1

**Table 2:** Venues for drug abuse.

Variable	*N*	%
Most important venue for taking drugs (*N* = 73)		
Own homes	41	56.2
Friends’ homes	15	20.5
Entertainment places	9	12.3
Public places	3	4.1
Workplaces	1	1.4
Hotels	2	2.7
Others	2	2.7

**Table 3:** Themes and sub-themes.

Themes	Sub-Themes	Cohen’s Kappa
The Current Trend of Drug Abuse	1. Switch from Large Discos to Private Parties	0.805
	2. Availability of and Accessibility to Drugs	0.815
	3. Psychological Safety When Taking Drugs Privately	1
The Structure of Social Networks and Social Capital	1. Drug Abusers’ Social Network via Bonding and Bridging Social Capitals	0.865
	2. Drug Supply Network via Linking Social Capital	1
	3. Mechanisms of Drug Transaction	1
	4. Trust as the Key to Enter the Networks	0.881

## References

[1] Narcotics Division Central Registry of Drug Abuse statistics; Security Bureau: HKSAR. https://www.nd.gov.hk/en/statistics_list.htm.

[2] Narcotics Division The Three-Year Plan on Drug Treatment and Rehabilitation Services in Hong Kong (2018–2020); Security Bureau: HKSAR. https://www.nd.gov.hk/pdf/three_year_plan_2018_2020_final_en.pdf.

[3] Task Force on Youth Drug Abuse Report of the Task Force on Youth Drug Abuse. http://www.legco.gov.hk/yr08-09/english/panels/se/papers/se1202-rpt081111-e.pdf.

[4] Lam C.W., Boey K.W., Wong A.O.O., Tse J.S.K. (2004). A Study of Substance Abuse in Underground Rave Culture and Other Related Settings.

[5] Narcotics Division (2016). Central Registry of Drug Abuse: Sixty-Sixth Report (2007–2016).

[6] Evans-Brown M., Sedefov R. (2017). New psychoactive substances: Driving greater complexity into the drug problem. Addiction.

[7] Hong Kong Council of Social Service Network on Substance Abuse Service: The Current Situations and Challenges of Drug Abuse in Hong Kong (Report No.: CB(2)2631/06-07/(01)). http://www.legco.gov.hk/yr06-07/chinese/panels/se/papers/secb2-2631-1-c.pdf.

[8] Lau J.T.F. (2013). Study on Drug Abuse Situation and Service Needs of Non-Engaged Youths in Hong Kong.

[9] Tam C.H., Kwok S.I., Lo T.W., Lam S.H.P., Lee G.K.W. (2018). Hidden drug abuse in Hong Kong: From social acquaintance to social isolation. Front. Psychiatry.

[10] Bourdieu P., Richardson J.G. (1986). The forms of capital. Handbook of Theory and Research for the Sociology of Education.

[11] Coleman J.S. (1988). Social capital in the creation of human capital. Am. J. Sociol..

[12] Grootaert C., Narayan D., Jones V.N., Woolcock M. Measuring Social Capital: An Integrated Questionnaire. https://openknowledge.worldbank.org/bitstream/handle/10986/15033/281100PAPER0Measuring0social0capital.pdf?sequence=1&isAllowed=y.

[13] Lin N. (2002). Social Capital: A Theory of Social Structure and Action.

[14] Coleman J.S. (1990). Foundations of Social Theory.

[15] Woolcock M. (2001). The place of social capital in understanding social and economic outcomes. Can. J. Policy Res..

[16] Babaei H., Ahmad N., Gill S.S. (2012). Bonding, bridging and linking social capital and empowerment among squatter settlements in Tehran, Iran. World Appl. Sci. J..

[17] Szreter S., Woolcock M. (2004). Health by association? Social capital, social theory, and the political economy of public health. Int. J. Epidemiol..

[18] Bolin K., Lindgren B., Lindström M., Nystedt P. (2003). Investments in social capital—Implications of social interactions for the production of health. Soc. Sci. Med..

[19] Glaeser E.L., Laibson D., Sacerdote B. (2002). The economic approach to social capital. Econ. J..

[20] Lundborg P. (2005). Social capital and substance use among Swedish adolescents: An explorative study. Soc. Sci. Med..

[21] Cohen S., Wills T.A. (1985). Stress, social support, and the buffering hypothesis. Psychol. Bull..

[22] Tsutsumi A., Tsutsumi K., Kayaba K., Igaraschi M. (1998). Health-related behaviours, social support and community morale. Int. J. Behav. Sci..

[23] May C.K. (2008). Drug courts: A social capital perspective. Sociol. Inq..

[24] Aldridge J., Askew R. (2017). Delivery dilemmas: How drug cryptomarket users identify and seek to reduce their risk of detection by law enforcement. Int. J. Drug Policy.

[25] Lovell A.M. (2002). Risking risk: The influence of types of capital and social networks on the injection practices of drug users. Soc. Sci. Med..

[26] Erickson P.G., Cheung Y.W. (1999). Harm reduction among cocaine users: Reflections on individual intervention and community social capital. Int. J. Drug Policy.

[27] Neaigus A. (1998). The network approach and interventions to prevent HIV among injection drug users. Public Health Rep..

[28] Latkin C.A., Forman V., Knowlton A., Sherman S. (2003). Norms, social networks, and HIV-related risk behaviors among urban disadvantaged drug users. Soc. Sci. Med..

[29] Valente T.W., Gallaher P., Mouttapa M. (2004). Using social networks to understand and prevent substance use: A transdisciplinary perspective. Subst. Use Misuse.

[30] Barman-Adhikari A., Begun S., Rice E., Yoshioka-Maxwell A., Perez-Portillo A. (2016). Sociometric network structure and its association with methamphetamine use norms among homeless youth. Soc. Sci. Res.

[31] Horne C. (2001). The enforcement of norms: Group cohesion and meta-norms. Soc. Psychol. Q..

[32] Fisher J. (1988). Possible effects of reference group-based social influence on AIDS risk behavior and AIDS prevention. Am. Psychol..

[33] Hall A., Wellman B., Cohen S., Syme S.L. (1985). Social networks and social support. Social Support and Health.

[34] Latkin C.A., Knowlton A.R., Hoover D., Mandell W. (1999). Drug network characteristics as a predictor of cessation of drug use among adult injection drug users: A prospective study. Am. J. Drug Alcohol Abuse.

[35] Scott J. (2012). Social Network Analysis.

[36] Teunissen H.A., Spijkerman R., Prinstein M.J., Cohen G.L., Engels R.C.M.E., Scholte R.H.J. (2012). Adolescents’ conformity to their peers’ pro-alcohol and anti-alcohol norms: The power of popularity. Alcohol. Clin. Exp. Res..

[37] Ennett S.T., Bauman K.E. (1993). Peer group structure and adolescent cigarette smoking: A social network analysis. J. Health Soc. Behav..

[38] Barrington C., Latkin C., Sweat M.D., Moreno L., Ellen J., Kerrigan D. (2009). Talking the talk, walking the walk: Social network norms, communication patterns, and condom use among the male partners of female sex workers in La Romana. Dominican Republic. Soc. Sci. Med..

[39] Latkin C.A., Kuramoto S.J., Davey-Rothwell M.A., Tobin K.E. (2010). Social norms, social networks, and HIV risk behavior among injection drug users. Aids Behav..

[40] Bright D.A., Delaney J.J. (2013). Evolution of a drug trafficking network: Mapping changes in network structure and function across time. Glob. Crime.

[41] Yeşilyurt H. (2014). The analysis of social capital and social networking of drug trafficking. J. Hum. Sci..

[42] Braun V., Clarke V. (2006). Using thematic analysis in psychology. Qual. Res. Psychol..

[43] Freelon D. (2010). ReCal: Intercoder reliability calculation as a web service. Int. J. Internet Sci..

[44] Neuendorf K.A. (2017). The Content Analysis Guidebook.

[45] Lloyd C. (2013). The stigmatization of problem drug users: A narrative literature review. Drugs Abingdon Engl..

[46] Putnam R.D. (2000). Bowling Alone: The Collapse and Revival of American Community.

[47] Lo T.W., Kwok S.I., Siegel D., van de Bunt H. (2012). Traditional organized crime in the modern world: How triad societies respond to socioeconomic change. Traditional Organized Crime in the Modern World: Responses to Socioeconomic Change.

[48] van Uhm D.P., Wong R.W. (2019). Establishing Trust in the Illegal Wildlife Trade in China. Asian J. Criminol..

[49] Stetina B.U., Jagsch R., Schramel C., Maman T.L., Kryspin-Exner I. (2008). Exploring hidden populations: Recreational drug users. Cyberpsychology.

[50] Bourdieu P. (1984). Distinction: A Social Critique of the Judgement of Taste.

[51] Bright D., Koskinen J., Malm A. (2019). Illicit network dynamics: The formation and evolution of a drug trafficking network. J. Quant. Criminol..

[52] Poplin D.E. (1979). Communities: A Survey of Theories and Methods of Research.

[53] European Monitoring Centre for Drugs and Drug Addiction (2007). 2007 Annual Report: The State of the Drugs Problem in Europe.

[54] Søgaard T.F., Kolind T., Haller M.B., Hunt G. (2019). Ring and bring drug services: Delivery dealing and the social life of a drug phone. Int. J. Drug Policy.

[55] Tzanetakis M., Kamphausen G., Werse B., von Laufenberg R. (2016). The transparency paradox. Building trust, resolving disputes and optimising logistics on conventional and online drugs markets. Int. J. Drug Policy.

[56] Schifano F., Deluca P., Baldacchino A., Peltoniemi T., Scherbaum N., Torrens M., Farre M., Flores M., Rossi M., Eastwood D. (2006). Drugs on the web; the Psychonaut 2002 EU Project. Prog. Neuropsychopharmacol. Biol. Psychiatry.

[57] Schifano F., Leoni M., Martinotti G., Rawaf S., Rovetto F. (2003). Importance of cyberspace for the assessment of the drug abuse market: Preliminary results from the Psychonaut 2002 EU Project. Cyberpsychol. Behav..

[58] Tam C.L., Foo Y.C. (2012). Contributory factors of drug abuse and the accessibility of drugs. Int. J. Collab. Res. Intern. Med. Public Health.

[59] European Monitoring Centre for Drugs and Drug Addiction (EMCDDA) and Europol (2013). EMCDDA–Europol Joint Report on a New Psychoactive Substance: 5-(2-Aminopropyl) Indole.

[60] Jackson L., Parker J., Dykeman M., Gahagan J., Karabanow J. (2010). The power of relationships: Implications for safer and unsafe practices among injection drug users. Drugs Abingdon Engl..

[61] Kirst M.J. (2009). Social capital and beyond: A qualitative analysis of social contextual and structural influences on drug use-related health behaviors. J. Drug Issues.

